# Valuable Natural Antioxidant Products Recovered from Tomatoes by Green Extraction

**DOI:** 10.3390/molecules27134191

**Published:** 2022-06-29

**Authors:** Mihaela Popescu, Petrica Iancu, Valentin Plesu, Maria Cristina Todasca, Gabriela Olimpia Isopencu, Costin Sorin Bildea

**Affiliations:** 1 Department of Chemical and Biochemical Engineering, University POLITEHNICA of Bucharest, 1, Gh. Polizu Street, Building A, Room A056, RO-011061 Bucharest, Romania; mihaela.popescu@chimie.upb.ro (M.P.); v_plesu@chim.upb.ro (V.P.); g_isopencu@chim.upb.ro (G.O.I.); sorin.bildea@upb.ro (C.S.B.); 2 Department of Organic Chemistry Costin Nenitescu, University POLITEHNICA of Bucharest, 1, Gh. Polizu Street, Building P, Room 014-015, RO-011061 Bucharest, Romania; cristina.todasca@upb.ro

**Keywords:** antioxidants, tomato oil, lycopene, β-carotene, ω fatty acids, supercritical CO_2_ extraction, green solvents, economic analysis

## Abstract

Lycopene, β-carotene and ω-fatty acids are major compounds in tomatoes with known antioxidant activity, capable of preventing health disorders. The identification of potential natural sources of antioxidants, extraction efficiencies and antioxidant activity assessments are essential to promote such products to be used in the food, pharmaceutical or cosmetic industries. This work presents four added-value products recovered from tomatoes: pigmented solid oleoresin, pigmented oil and two raw extracts from supercritical and Soxhlet extraction. Different parameters including the matrices of tomatoes, extraction methods, green solvents and operating parameters were varied to obtain extracts with different qualities. Extract analysis was performed using UV–VIS, FT–IR, GC–MS, Folin–Ciocalteu and DPPH methods. The highest-quality extract was the solid oleoresin obtained from pomace using supercritical CO_2_ extraction at 450 bar, 70 °C and 11 kg/h: 1016.94 ± 23.95 mg lycopene/100 g extract, 154.87 ± 16.12 mg β-carotene/100 g extract, 35.25 ± 0.14 mg GAE/g extract and 67.02 ± 5.11% inhibition DPPH. The economic feasibility of the three extraction processes (1:10:100 kg dried pomace/batch as scalability criterion) was evaluated. The most profitable was the supercritical extraction process at the highest capacity, which produces pigmented solid oleoresin and oil with high content of lycopene valorized with a high market price, using natural food waste (pomace).

## 1. Introduction

Tomatoes belong to the group of the most consumed vegetables in the world, due to the easy methods of cultivation and to the benefits they bring to human health through their important bioactive compounds distributed in the peels and seeds [1,2]. Tomato peels contain carotenoids (mostly lycopene and β-carotene), polyphenol compounds such as flavonols (quercetin, kaempferol, myricetin), flavonol glycosides (rutin), flavanones (naringenin chalcone) and hydroxycinnamic acids (chlorogenic, caffeic, p-coumaric and ferulic acids) and minerals (potassium, calcium, sodium, magnesium). Phenolic compounds are located in the dermal tissues of tomatoes to protect against radiation, pathogens and predators. The phenolic content in tomato parts is the highest in the peels, being followed by the seeds and the pulp [3,4]. Tomato seeds contain 17–23% oil rich in important fatty acids (linoleic, oleic, palmitic, stearic and linolenic acids), vitamins (α-, β-tocopherols), amino acids (glutamic and aspartic acids, lysine, arginine, valine, leucine), proteins (globulin, albumin, gliadin, glutenin) and phytosterols [5]. However, the most important classes of bioactive compounds contained by tomatoes are carotenoids and ω-fatty acids [5,6]. Carotenoids are pigments with anti-inflammatory, anti-radical and antioxidant activities. They are authorized additives used in foods, cosmetics or supplements to enhance the nutritional value [7,8,9], flavor or color [10]. Carotenoid consumption in Middle and Eastern Europe in 2017 was 448.4 metric tons, with 13.3 metric tons in Romania. For 2022, an increase of around 11% is expected, in Romania reaching up to 13.9 metric tons [11]. This increase in carotenoid consumption is given by the awareness of the impact that food has on human health [12]. The main carotenoids found in tomato extracts are lycopene and β-carotene. Lycopene accounts for 80–90% [13,14] and has the highest antioxidant activity, radical scavenging capacity and singlet oxygen quenching ability of all carotenoids [1,6] due to its long conjugated double bond system [13]. In addition to their antioxidant properties, lycopene and β-carotene have also coloring power, covering a range of yellow–orange–red colors [11]. The human body cannot synthetize these pigments, so the only source is achieved through the diet, from synthetic or natural sources [15]. Lycopene has the food additive number E160d [10], while β-carotene has E160a [11]. They are used as natural antioxidant preservatives [16] and coloring agents in milk products, condiments, sauces, spreads, alcoholic [11] and non-alcoholic drinks [15], meat products [14], butter, mayonnaise, ice cream [10], bread [3], baked goods [17], in cosmetic formulations such as creams or body butters [18] or in nutraceutical supplements [11]. Fatty acid groups found in tomato seed oil are saturated palmitic (C16:0) and stearic (C18:0) acids, monounsaturated oleic acid (C18:1ω9) and polyunsaturated fatty acids as linoleic (C18:2ω6) and linolenic (C18:3ω3) acids. Between these compounds, only linoleic and linolenic acids cannot be synthesized by the human body. The only source is through the diet, especially from edible plant oils [19]. The major fatty acid found in tomato seed oil is ω6-linoleic acid, this being followed by ω9-oleic acid. The high composition of unsaturated fatty acids makes tomato seed oil an important high-nutritional-quality edible oil with health benefits in the prevention of cardiovascular and atherosclerosis diseases. Moreover, it was found that tomato seed oil has higher antioxidant activity than common edible oils, such as olive, sunflower and soybean oils [20]. Thus, tomato is a potential source of carotenoids and essential fatty acids with applications in the food, cosmetic and pharmaceutical industries. The tendency is to use food additives obtained from natural crops, because the occurrence of many diseases is associated with the consumption of foods rich in synthetic additives [9,12,16]. This requires accessible plant raw materials and an efficient and environmentally friendly extraction method.

The annual production of tomatoes worldwide is approximately 180 million tons, of which more than a quarter is used for processing [21]. Tomato pomace is a residue provided by the tomato industry and consists of around 1.5–30% [2,10,21] of the tomato fresh weight. It contains a mixture of pulp residue, peels and seeds, undesired in tomato products [2], their amount and distribution varying with tomato varieties [6]. The amount of this waste is large and has a negative impact on the environment due to its handling and disposal issues [22,23]. Despite this, it contains a cocktail of antioxidant compounds, such as carotenoids, polyphenols and minerals, and 20–40% high-quality oil rich in amino acids, protein, fatty acids and vitamins [24,25]. Currently, this by-product, industrial or domestic, is either disposed of, used in agriculture [1] or used as an additive in animal food formulations. Processing and recovery of this low-cost and underestimated residue would solve several problems, such as waste disposal and the introduction of a new feedstock for natural lycopene and tomato oil [6,7].

Carotenoids and oils can be isolated from plant matrices by extraction. To be used in the health and food industry, this process must be environmentally friendly, efficient and economically reliable. Numerous extraction techniques have been analyzed over time to extract carotenoids from plant materials, both traditional, such as maceration and solvent extraction, as well as more complex methods, such as microwave- or ultrasound-assisted extraction [12,16], accelerated solvent extraction, pulsed electric-field assisted extraction [25], supercritical fluid extraction and enzyme- or surfactant-assisted extraction [12,16,26]. Each of these methods has advantages and limitations, the choice of the extraction method being made after a broad analysis of what is desired to be obtained, along with certain compromises. The most common issues in carotenoid extraction are their stability and their solubility in solvents [7,8], while other characteristics are the toxicity of the extract, the environmental impact, the target compounds’ purities and the extraction method. Carotenoids are lipophilic compounds [9,12], insoluble in polar solvents such as water, slightly soluble in medium-polarity solvents such as ethanol, acetone, dichloromethane or ethyl acetate and highly soluble in non-polar solvents such as hexane, chloroform or edible vegetable oils [25,27]. The polarity values of these solvents are presented in Table 1.

Nowadays, there is a great interest in using environmentally friendly industrial processes, able to preserve the biological properties of the extracts. As a consequence, many efforts are made to improve extraction methodologies in order to avoid the toxic effects and to reduce the energy consumption. Thus, green solvents produced from natural resources, ionic liquids [13,25] and supercritical fluids [28] have emerged.

Soxhlet extraction (SE) is a well-established method [26] characterized by high extraction yields, with important disadvantages related to the toxicity and purity of the extract, the degradation of solutes by oxidation reactions and environmental issues caused by high solvent consumption and waste production [1,9]. The main drawback of this method is that the purification of the extract demands expensive purification processes [12,29]. However, using approved green solvents for the food industry, such as bioethanol or ethyl acetate [9,17,28], SE becomes a greener method.

The supercritical fluid extraction (SFE) process is a green extraction method [12] that is promoted because it uses non-toxic solvents, reducing the energy consumption; it results in a clean extract without the need for purification [14] and degradation reactions are avoided due to the absence of light and air [25]. Supercritical fluids are solvents with temperature and pressure above their critical values, having superior thermodynamic properties, such as solvent power and mass transfer rates, to conventional solvents [1]. Carbon dioxide (CO_2_) is the most used supercritical solvent because it has moderate critical conditions (32.1 °C and 73.8 bar), having the ability to extract thermo-sensitive solutes, mainly non-polar or moderately polar. It is cheap and it shows the main characteristics of a green solvent, such as non-toxicity, biodegradability and recyclability [28]. For high performance of the SFE process, the most important step involves the optimization of the operation conditions [23,25]. The main extraction parameters in carotenoid recovery by SFE are the characteristics of the sample, such as moisture, particle size and the part of the plant [28], and characteristics of the process through extraction conditions such as pressure, temperature, time, CO_2_ flow rate, addition, type and percentage of co-solvent or modifier [14].

The aim of this work is to extract and evaluate natural value-added products from tomatoes using green extraction technologies at laboratory scale, coupled with the economic aspects of scale-up processes. Extraction process efficiencies are evaluated based on the effects of different factors such as tomato matrices (slices and pomace), extraction methods (Soxhlet and supercritical fluid extraction), green solvents (bioethanol, ethyl acetate and carbon dioxide), sets of operating parameters (pressure and flow rate for supercritical process) and product consistency (raw extract, oil, oleoresin). Extraction efficiency, carotenoids and fatty acid profiles, total phenolic content and antioxidant activities of tomato extracts are compared. An economic analysis is performed for different scale-up capacities to produce identified valuable extracts to gain profit.

## 2. Results and Discussion

### 2.1. Preparation of Tomato Samples

The water content in fresh tomato samples acts as a barrier for carotenoid extraction (mainly in the SFE method), preventing the dissolution of solutes in the solvents. Therefore, the water from tomato samples was removed. To prepare 1.2 kg dried tomato slices (TS), 220 tomato pieces with medium size were used. For the same quantity of dried tomato pomace (TP), a ten times higher mass of tomatoes was needed. For tomato seed (S) preparation, 39 kg of fresh tomatoes were needed (Table 2).

**Figure 1:**
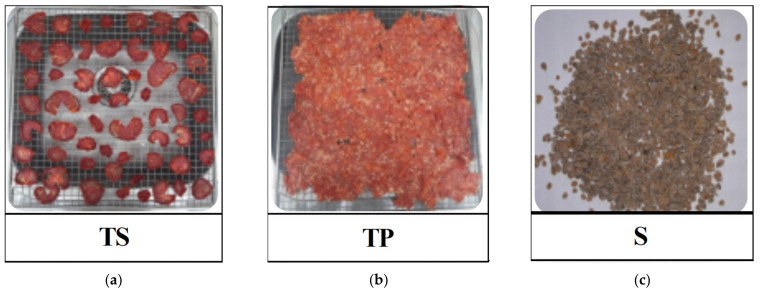
Dried tomato samples: (**a**) tomato slices (TS); (**b**) tomato pomace (TP); (**c**) tomato seeds (S).

Prepared TS and TP fresh samples’ moisture expressed on a wet basis were 93.57% and 93.67%, respectively. These results are confirmed by other studies with wet basis moistures around 93.22–95.14% for fresh tomatoes as TS [30] and 63–92.7% for TP [3]. The seed content of the samples was also determined by gravimetric analysis. TP contained 22–28% seeds, while TS contained 12–17% seeds. Tomato seeds act as a modifier, ensuring a certain amount of oil in the extraction process, improving the extraction efficiency [23]. The amount of tomato seeds (S) needed for the oil recovery was obtained from 4 kg of tomato pomace. Dried tomato samples TS and TP are presented in Figure 1.

### 2.2. Extraction Efficiency

Five types of extracts, which differed in consistency, were obtained from TP and TS samples. Raw extracts (SE) with the consistency of solid oleoresins were obtained by Soxhlet extraction using green solvents such as bioethanol (1) or ethyl acetate (2). By supercritical fluid extraction, using two sets of operating parameters differing in extraction pressure and green solvent (carbon dioxide) flow rate as 400 bar and 9 kg/h (set 1) and 450 bar and 11 kg/h (set 2), raw extracts (SFE) with the consistency of liquid oleoresins were recovered. By centrifuging SFE raw extracts, three other products were separated as fraction A with the consistency of a pigmented oil rich in carotenoids, fraction B with the consistency of a pigmented solid oleoresin rich in carotenoids and fraction C with the consistency of a liquid. Moreover, tomato seed oil was extracted with hexane by the Soxhlet method. The extraction yields for each experiment are presented in Table 3. Three groups of extraction yields (seeds, slices and pomace) were analyzed applying Hartley’s F_max_ test to verify the homogeneity of variances for individual groups. All groups of measured extraction yields presented homogeneity of variance, at the level of significance α = 0.05.

#### 2.2.1. Soxhlet Extraction (SE)

**Figure 2:**
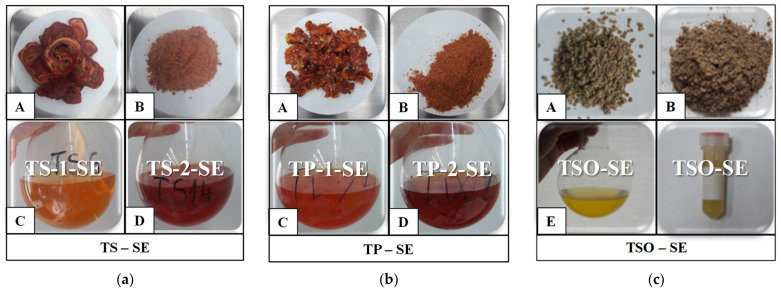
Tomato samples and raw extracts obtained with Soxhlet extraction (SE) using extraction solvents (1) bioethanol and (2) ethyl acetate, A—samples before grinding, B—samples after grinding, C—bioethanol extract (1), D—ethyl acetate extract (2), E—hexane extract before and after evaporation of the solvent: (**a**) tomato slices (TS); (**b**) tomato pomace (TP); (**c**) tomato seeds (S).

The SE method’s efficiency was checked analyzing two factors, solvent affinity and the seed content of the samples. From Figure 2a,b, it can be observed that raw extracts obtained with bioethanol from both types of samples (TS-1-SE, TP-1-SE) were less pigmented, with orange colors, than raw extracts with ethyl acetate (TS-2-SE, TP-2-SE), with red colors, regardless of sample type. The intense red color is associated with a higher carotenoid content [31]. For both types of tomato samples, the extraction was improved when ethyl acetate (2) was used, with 40% for TS and 8% for TP. Other studies also demonstrated that ethyl acetate is a better extracting solvent for carotenoids than bioethanol [13,29] and even than hexane [9]. Due to the different contents in the seeds of tomato samples and different affinity of both solvents, the calculated extraction efficiencies were between 5.92 ± 0.69 and 13.23 ± 1.14 g extract/100 g dried tomato slices using solvent (1) and 8.72 ± 0.93–14.33 ± 1.19 g extract/100 g dried tomato pomace using solvent (2). These results are similar to other studies: 8.46% from TP and 4.41% from tomato peels [1] or 6.5–19.3% from TP [7]. For tomato seed oil (TSO-SE), the extraction yield was 20.21 ± 1.22 g oil/100 g dried tomato seeds. This value falls within the range reported in the literature of 19–23% in terms of the oil content of tomato seeds recovered using solvent extraction [32].

#### 2.2.2. Supercritical CO_2_ Extraction (SFE)

**Figure 3:**
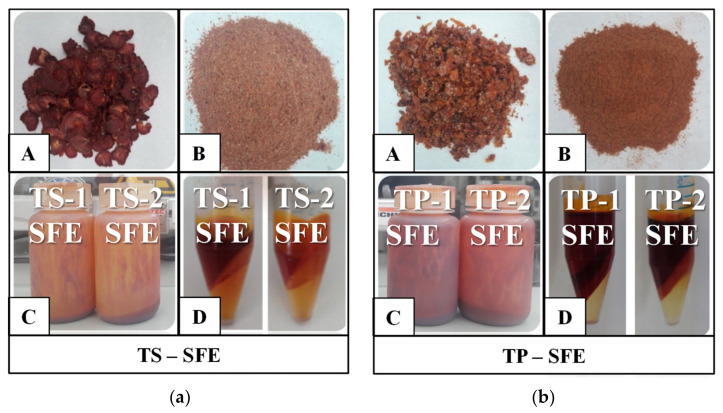
Tomato samples, raw extracts and centrifuged fractions obtained with supercritical CO_2_ extraction (SFE) using operating parameter set (1) at 400 bar, 70 °C, 9 kg/h and set (2) at 450 bar, 70 °C, 11 kg/h. A—before grinding, B—after grinding, C—operating parameter sets raw extracts (1,2), D—operating parameter sets centrifuged extracts (1,2): (**a**) tomato slices (TS); (**b**) tomato pomace (TP).

The seed content of the samples, the extraction pressure and the solvent flow rate were considered as factors that can affect the quality of raw extract obtained with SFE. Tomato samples subjected to SFE and extracts are presented in Figure 3.

SFE raw extracts are red and extracts obtained from pomace are more pigmented. Regarding the operating parameters, set (2) raw extracts had more intense colors. The extraction yield is improved when the peel/seed ratio is lower. In the SFE process, the yield is increasing with pressure due to the improvement in the supercritical CO_2_ density related to the solvating power, which favors the solubilization of target compounds. Moreover, higher CO_2_ rates lead to higher extraction yields [28]. By using operating parameter set (2) at high values of pressure (450 bar) and CO_2_ flow rate (11 kg/h), the extraction was more efficient than operating parameter set (1), regardless of the tomato sample (Table 3). Yields were improved with 21% for TS and 19% for TP. Obtained yields were between 5.25 ± 0.79 and 6.64 ± 1.12 g extract/100 g dried TS and between 10.02 ± 1.14 and 12.35 ± 1.55 g extract/100 g dried TP. These values are in line with previous results reported by other authors: 10.3–13.4% from TP [23], 11.4–24.6% from TP [7], 12.51% from TP and 2.5% from peels [1]. An increase of 100% for extraction efficiency was obtained for pomace samples, regardless of the operating parameter sets.

#### 2.2.3. Extract Centrifugation

The consistency of the raw extracts from SFE is similar to a liquid oleoresin. For an accurate analysis, separation of the raw extracts’ phases was necessary to evaluate the quality of the products. Using the centrifugation method, three fractions were obtained. The upper fraction (SFE-A) was red and oily, the middle fraction was a dark red solid oleoresin (SFE-B) and the lower fraction was a yellow liquid (SFE-C) (Figure 3). The consistency of SFE-B fractions was similar to SE raw extracts. Longo, Leo and Leone [31] and Vallecilla-Yepez and Ciftici [7] applied the same centrifugation step on SFE tomato extracts and obtained two fractions: one red and oily fraction as SFE-A and one insoluble, crystalline pellet fraction as SFE-B. 

TS raw extracts contained 66–73% SFE-A, 5–7% SFE-B and 20–30% SFE-C fractions, while TP raw extracts had 73–77% SFE-A, 13–15% SFE-B and 11–13% SFE-C. TP raw extracts were more pigmented and contained higher amounts of SFE-A and SFE-B fractions than TS raw extracts. SFE-A and SFE-B fractions were abundant in oil and carotenoids; thus, it is expected that TP raw extracts will have higher amounts of these fractions due to the higher content of tomato seeds.

### 2.3. Qualitative and Quantitative Analysis of SE and SFE Raw Extracts

#### 2.3.1. Carotenoid Qualitative Analysis

The quality of tomato raw extracts was assessed using the UV–VIS spectrometry method based on the presence of specific shapes and peaks of the main analyzed carotenoids, lycopene and β-carotene.

**Figure 4:**
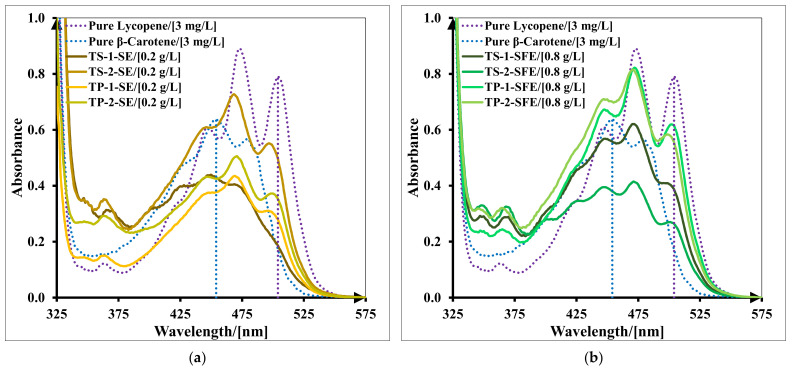
UV–VIS spectra of tomato raw extracts: (**a**) SE raw extracts obtained from tomato slices (TS) and tomato pomace (TP), using as extraction solvents (1) bioethanol and (2) ethyl acetate; (**b**) SFE raw extracts obtained from tomato slices (TS) and tomato pomace (TP), using as operating parameters set (1) at 400 bar, 70 °C, 9 kg/h and set (2) at 450 bar, 70 °C, 11 kg/h.

Pure lycopene, pure β-carotene, SE and SFE raw extracts’ spectra in acetone/hexane (*v*/*v*, 1:1) are presented in Figure 4. The maximum absorption peaks of pure lycopene are at 447 nm, 473 nm and 504 nm wavelengths, while for β-carotene, they are at 426 nm, 454 nm and 480 nm [33]. All the raw extracts presented specific shapes of carotenoids, with three peaks, at lycopene and β-carotene’s maximum absorption wavelengths. SE raw extracts obtained with bioethanol (1) had lower absorbance values than ethyl acetate (2) raw extracts. This shows that there are differences between the amounts of carotenoids extracted by these solvents. Moreover, a difference in the absorbance values of extracts obtained by SFE for both operating parameter sets was observed. SE raw extracts were more concentrated in carotenoids than SFE, similar absorbance values being reached at different concentrations, namely 0.2 g/L for SE and 0.8 g/L for SFE raw extracts.

#### 2.3.2. Carotenoid Content

**Figure 5:**
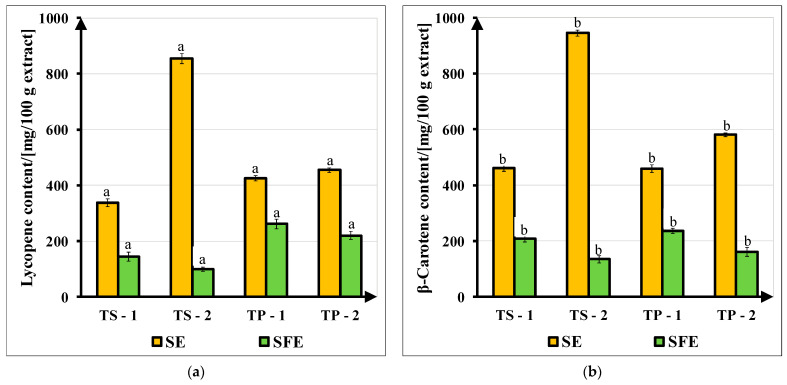
Carotenoid content of SE and SFE raw extracts obtained from tomato slices (TS) and tomato pomace (TP), using as extraction solvents (1) bioethanol and (2) ethyl acetate and as operating parameters set (1) at 400 bar, 70 °C, 9 kg/h and set (2) at 450 bar, 70 °C, 11 kg/h: (**a**) lycopene; (**b**) β-carotene. Different superscripts (**a**,**b**) indicate statistically different carotenoid concentration (*p* < 0.05) at a level of significance α = 0.05.

Lycopene and β-carotene contents (mg carotenoid/100 g extract) are shown in Figure 5. SE raw extracts contained higher amounts of both carotenoids than SFE raw extracts, regardless of the used extraction solvent (in SE) and operating parameters (in SFE). Lycopene contents of SE and SFE raw extracts varied between 336.77 ± 14.05 and 854.50 ± 7.51 mg/100 g extract and between 99.41 ± 5.72 and 261.70 ± 6.66 mg/100 g extract, respectively. Calculated β-carotene contents were between 459.06 ± 6.46 and 945.00 ± 10.87 mg/100 g extract and between 134.77 ± 13.28 and 236.11 ± 10.17 mg/100 g extract. Calculated values of carotenoid concentrations presented homogeneity of variances (*p* < 0.05), checked with Hartley’s F_max_ test, at a significance level of α = 0.05. These carotenoid compositions varied with the extraction method (SE, SFE), the type of tomato sample (TS, TP), extraction solvent (1, 2) or operating parameter set (1, 2). Extracts from SE were richer in carotenoids than those obtained from SFE, although these had traces of solvent.

Extraction solvents influence the carotenoids’ recovery due to their polarity. The descending order of the used green solvents’ polarity is bioethanol > ethyl acetate > carbon dioxide [27]. Ethyl acetate (2) raw extracts had higher content of both lycopene (854.50 ± 7.51 mg/100 g extract from TS and 454.64 ± 8.76 mg/100 g extract from TP) and β-carotene (945.00 ± 10.87 mg/100 g extract from TS and 580.96 ± 9.51 mg/100 g extract from TP) than bioethanol (1).

For the SFE method, higher carotenoid contents were obtained using operating parameter set (1) at lower values of pressure and CO_2_ flow rate. The same behavior was observed by Kehili et al. [13], who found lower yields at lower values of pressure and CO_2_ flow rate. Similar results were reported by Romano et al. [1], with 341 mg lycopene/100 g extract and 470 mg β-carotene/100 extract from TP-SE extracts or 61 mg lycopene/100 extract and 47 mg β-carotene/100 extract from TP-SFE extracts. Comparable values were also reported by Vallecilla-Yepez and Ciftici [7], who obtained 70–240 mg lycopene/100 g extract from TP-SE extracts and 20–260 mg lycopene/100 g extract from TP-SFE extracts. However, a higher extraction yield does not ensure higher recovery of carotenoids, mostly for SFE extracts. Moreover, some unexpected discrepancies in the quantification of carotenoids can appear due to the variety of tomato samples and to the heterogeneity of tomato extracts [33]. TP raw extracts had a higher amount of oil; hence, the extracted carotenoids were dissolved in a larger amount of liquid than the carotenoids extracted from TS, which led to an extract diluted in tomato seed oil.

### 2.4. Qualitative and Quantitative Analysis of SFE Fractions

The composition of SFE fractions was evaluated using UV–VIS and FT–IR analyses. Pure lycopene and β-carotene and tomato seed oil spectra were used as references in the quality determination of SFE fractions, checking the spectra’s similarities. Due to the similarities between samples, both methods were applied on TP-2-SFE extract fractions.

#### 2.4.1. Carotenoid Qualitative Analysis Using UV–VIS Method

**Figure 6:**
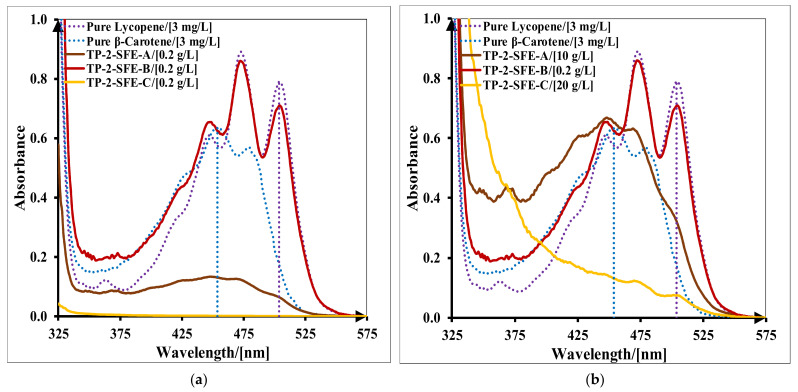
UV–VIS spectra of SFE fractions (SFE-A, SFE-B, SFE-C) obtained from tomato pomace (TP) with operating parameter set (2) at 450 bar, 70 °C and 11 kg/h (TP-2-SFE): (**a**) fraction concentrations (SFE-A—0.2 g/L, SFE-B—0.2 g/L, SFE-C—0.2 g/L); (**b**) fraction concentrations (SFE-A—10 g/L, SFE-B—0.2 g/L, SFE-C—20 g/L).

UV–VIS spectra of separated fractions of SFE extract (TP-2-SFE), obtained from tomato pomace (TP) with operating parameter set (2), are presented in Figure 6. At 0.2 g/L concentration of sample, only the SFE-B fraction presented specific shapes of carotenoids (mostly lycopene), the spectrum being clear, with three peaks (Figure 6a).

For a conclusive analysis of the spectra shapes, appropriate concentrations of the fractions were chosen, as 10 g/L for the SFE-A fraction and 20 g/L for the SFE-C fraction (Figure 6b). At these concentrations, SFE-A fraction presents specific carotenoids peaks (mostly β-carotene), with a less defined spectrum. However, it also contained lycopene, its presence being indicated by the shoulder of the spectrum at 504 nm. The SFE-C fraction had some specific carotenoid peaks, but, upon analyzing their concentrations, their content was probably in trace amounts. Similar distributions and proportions of carotenoids in centrifuged fractions of TS-1-SFE, TS-2-SFE and TP-1-SFE extracts were obtained.

#### 2.4.2. Carotenoid Qualitative Analysis Using FT–IR Method

**Figure 7:**
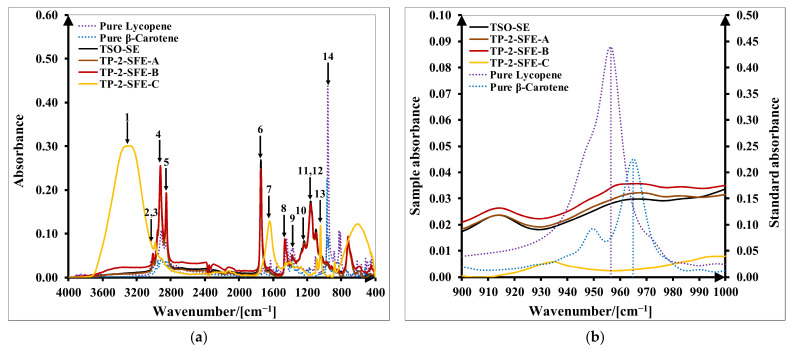
FT–IR spectra of SFE fractions (SFE-A, SFE-B, SFE-C) obtained from tomato pomace (TP) with operating parameter set (2) at 450 bar, 70 °C and 11 kg/h (TP-2-SFE): (**a**) 400–4000 cm^−1^ region; (**b**) 900–1000 cm^−1^ region.

The FT–IR method was also used to identify the components of all separated fractions, based on spectral information. The determination of the main functional groups is based on the presence of specific typical absorption bands. Specific absorption bands of lycopene in the FT–IR spectrum were found at 3285 cm^−1^ (C-H stretching), 2856–2924 cm^−1^ (C-H symmetrical and asymmetrical stretching), 1643 cm^−1^ (vibrational C=C stretching), 1444 cm^−1^ (C-H bending and vibrational symmetric of CH_2_), 1370 cm^−1^ (vibrational CH_3_), 1156 cm^−1^ (C-C stretching), 1082 cm^−1^ (CH_3_ attached with polyene system) and 956–960 cm^−1^ (R–CH=CH–R bending out of plane) [34,35,36]. Unique markers for lycopene and β-carotene were seen at 956–960 cm^−1^ and 965–968 cm^−1^ [35,37,38], respectively. Tomato seed oil’s specific functional groups assigned to fatty acids and their esters were noted at 3008–3010 cm^−1^ (C–H stretching), 2854–2924 cm^−1^ (C–H symmetrical and asymmetrical stretching), 1745–1746 cm−1 (C=O stretching), 1464–1465 cm^−1^ (C-H bending), 1238 cm^−1^ (C-O stretching) and 1162–1163 cm^−1^ (C-H bending), 1097–1118 cm^−1^ (C-O stretching) [39,40]. Spectra of pure lycopene, β-carotene and tomato seed oil (TSO-SE) presented in Figure 7 are in concordance with the literature results. These spectra were compared to check the similarities with the SFE fractions.

TP-2-SFE fractions contain 14 absorption peaks assigned to different functional groups, as presented in Figure 7a and Table 4. SFE-A and SFE-B fractions present specific absorption bands characteristic of carotenoids and tomato seed oil. The difference between these fractions is that TP-2-SFE-B presents lycopene and β-carotene absorption bands (958–974 cm^−1^), while TP-2-SFE-A seems to contain mostly β-carotene, because an absorption band is found at 963–973 cm^−1^. Moreover, from Figure 7b, it can be observed that the TP-2-SFE-B fraction’s absorbances are higher than TP-2-SFE-A. The TP-2-SFE-C fraction contains specific bands for O-H stretching (3000–3600 cm^−1^) and O-H bending (1500–1700 cm^−1^), specific to water and ethanol [37]. The ethanol content of the TP-2-SFE-C fraction results from the washing step of the plant among the SFE extraction experiments. Thus, the contribution of the SFE-C fraction was not considered for carotenoids’ quantitative analysis. Similar distributions and proportions of centrifuged fractions of TS-1-SFE, TS-2-SFE and TP-1-SFE extracts in terms of carotenoids and tomato seed oil positions and specific spectral bands were obtained.

#### 2.4.3. Carotenoid Content

**Figure 8:**
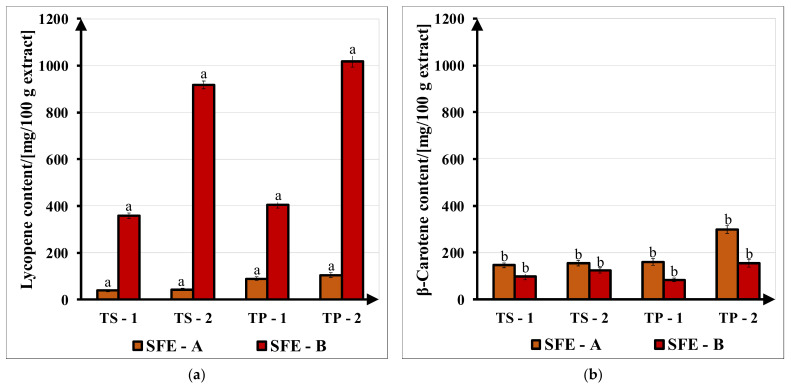
Carotenoid content of SFE fractions (SFE-A and SFE-B) obtained from tomato slices (TS) and tomato pomace (TP) with operating parameter set (1) at 400 bar, 70 °C, 9 kg/h and set (2) at 450 bar, 70 °C and 11 kg/h: (**a**) lycopene; (**b**) β-carotene. Different superscripts (**a**,**b**) indicate statistically different carotenoid concentration (*p* < 0.05) at a level of significance α = 0.05.

The carotenoid content of SFE fractions in terms of lycopene and β-carotene content, obtained through the UV–VIS method, is presented in Figure 8. The lycopene content of the SFE-A and SFE-B fractions was between 38.74 ± 0.18–105.02 ± 0.40 mg/100 g for the SFE-A fraction and 359.66 ± 1.84–1016.94 ± 8.95 mg/100 g for the SFE-B fraction. The results for β-carotene were between 146.24 ± 4.95–299.06 ± 5.64 mg/100 g for the SFE-A fraction and 83.96 ± 8.04–154.87 ± 6.12 mg/100 g for the SFE-B extract. SFE-B fractions had higher carotenoid contents than SFE-A fractions and their values are in line with the SE extracts.

Regarding operating parameters, although the carotenoid content in SFE raw extracts obtained with set (1) was higher, analyzing SFE-B fractions, it seems that set (2) was more efficient, regardless of the type of tomato sample. Carotenoid yields are positively correlated with pressure and flow rate, these parameters playing a significant role for carotenoid recovery with SFE [7,23,25]. For the carbon dioxide solvent, at lower pressures (set 1), the polarity of supercritical CO_2_ is comparable to hexane, while, at high pressures (set 2) with chloroform, carotenoids are more soluble in chloroform than in hexane [8]. The obtained results are in line with Longo, Leo and Leone’s [31] study, which reported 306 mg lycopene/100 g oily fraction, 582 mg lycopene/100 g insoluble fraction, 80 mg β-carotene/100 g oily fraction and 55 mg β-carotene/100 g insoluble fraction from tomato pulp oleoresin with the SFE method. Similar values of 300–380 mg lycopene/100 g oily fraction and 150–330 mg lycopene/100 g insoluble fraction separated from TP-SFE extracts were reported in [7]. Carotenoid distribution in tomato extracts was also analyzed. Lycopene/β-carotene mean ratios from TS in SE and SFE raw extracts were 45/55 and 42/58. In SFE-A and SFE-B fractions, these ratios were 22/78 and 83/17, respectively. For TP in SE and SFE raw extracts, mean ratios were 46/54 and 55/45, while from SFE-A and SFE-B fractions, their values were 43/57 and 85/15. These ratios show that lycopene content is higher in SFE-B fractions and SE raw extracts, which are not diluted by oil, as in SFE raw extracts and SFE-A fractions. Regarding extraction parameters, higher ratios were obtained using ethyl acetate (2) in SE and operating parameter set (2) in SFE. Comparable values of lycopene/β-carotene ratios were presented in the literature: 78/22 from tomato pulp oleoresin [31], 73/27 in dried TP [26] and 93/7 in dried TP [8].

#### 2.4.4. FAME Content

Fatty acid methyl ester (FAME) profiles of TSO-SE are presented in Table 5. The TSO-SE composition is in agreement with results reported in the literature as 12.26–14.42% palmitic acid, 3.59–5.15% stearic acid, 17.33–27.76% oleic acid and 53.70–64.63% linoleic acid [5,19,20]. The major fatty acid found in TSO-SE was ω6-linoleic acid of 55.59 ± 0.12%, being followed by ω9-oleic acid of 23.41 ± 0.56%.

The composition and proportion of the peel/seed ratio influence both the extraction yield and the recovery of target compounds. Thus, carotenoid recovery through extraction is dependent on the type of tomato sample, the type of extraction solvent in SE, type of operating parameter set in SFE and the composition of the extract/fraction through the oil content. This oil contains mainly ω6 and ω9 fatty acids, which give this product a superior quality for use in the food industry.

### 2.5. Total Phenolic Content of Tomato Raw Extracts

Total phenolic contents of SE and SFE tomato raw extracts determined by the Folin–Ciocalteu method are presented in Table 6. Higher values of phenolic content were found for TP extracts, regardless of the extraction solvent in SE or operating parameters in SFE. TP samples were rich in peels and seeds, while TS samples were abundant in pulp residue. However, the extraction method also influences the phenolic content of the obtained extract. For SE raw extracts, values ranged between 17.32 ± 0.08 and 30.91 ± 0.14 mg GAE/g extract, while for SFE raw extracts, they were between 22.59 ± 0.06 and 35.25 ± 0.14 mg GAE/g extract. Calculated values of total phenolic content presented homogeneity of variances (*p* < 0.05), checked with Hartley’s Fmax test at α = 0.05 level of significance. These contents were comparable to those reported by Romano et al. [1] as 2.22–5.79 mg GAE/g extract with the SE method and 22.63–78.32 mg GAE/g extract with the SFE method from tomato peels and pomace. Higher phenolic contents were obtained with the SFE method against the SE method, regardless of the type of sample. Phenolic compounds are polar compounds concentrated in hydrophilic fractions [24] and bioethanol is more polar than ethyl acetate [26]; thus, bioethanol extracts (TS-1-SE and TP-1-SE) are more abundant in phenolic compounds. This behavior was observed also in Abassi et al.’s [4] study, who reported the highest phenolic content from peels and seeds using ethanol against ethyl acetate as extraction solvents in the SE method. On the other hand, although carbon dioxide is a non-polar compound, when extraction parameters such as pressure or temperature are optimized, the polarity of this solvent is modified and the efficiency for polar compound recovery is increased [41,42].

### 2.6. Antioxidant Activity of Tomato Samples

The antioxidant activity of tomato extracts was determined in terms of the ability of the antioxidant compounds found in the tomato extracts to inhibit oxidation. DPPH quenching ability (%) values of tomato samples are presented in Table 6. Responsible for the antioxidant activity in tomatoes are phenolic compounds in the hydrophilic fraction and tocopherols, lycopene and β-carotene and fatty acids in the lipophilic fraction, because all compounds act synergically against DPPH free radicals [1,4,13]. The antioxidant activity values of SE raw extracts were between 27.61 ± 1.26 and 43.35 ± 3.36%, being lower than 37.30 ± 3.25–47.59 ± 4.05% for SFE raw extracts. The same behavior was reported in the literature on dried tomato powder, where the SFE extract quality was by far superior to SE extracts [31]. The variances calculated for the compared groups of antioxidant activities (slices and pomace) did not differ in a statistically significant manner.

For raw extracts, higher antioxidant activities of 40.78 ± 3.41–43.35 ± 3.36% were obtained with bioethanol (1) in SE and 39.26 ± 3.02–47.59 ± 4.01% with operating parameter set (1) in SFE. However, SFE fractions had higher antioxidant activities than raw extracts. The highest values were achieved from fractions separated from SFE raw extracts with operating parameter set (2). The antioxidant activities of SFE-A fractions varied between 38.41 ± 3.04 and 49.65 ± 4.21%, while for SFE-B fractions, obtained values were between 29.89 ± 2.05 and 67.02 ± 5.11%. These are lipophilic fractions, where the antioxidant activity is mainly influenced by the carotenoids and the fatty acid content. When the tomato sample type was analyzed, TP extracts had higher antioxidant activities than TS extracts. The highest antioxidant activity of 67.02 ± 5.11% was for the TP-2-SFE-B fraction, enriched in carotenoids. Comparable values of 36–38% were presented by Shahzad et al. [43] from whole tomatoes, while Kehili et al. [13] obtained 38–86% from tomato peels.

### 2.7. Economic Analysis

To estimate the extraction process profitability, three plant capacities were analyzed to produce four valuable extracts with antioxidant activity using dried tomato pomace as a raw material: SE raw extract (TP-2-SE), SFE raw extract (TP-2-SFE), pigmented solid oleoresin concentrated in lycopene (TP-2-SFE-B) and pigmented oil extract rich in carotenoids (TP-2-SFE-A). Annual manufacturing costs and profits are presented in Table 7.

**Figure 9:**
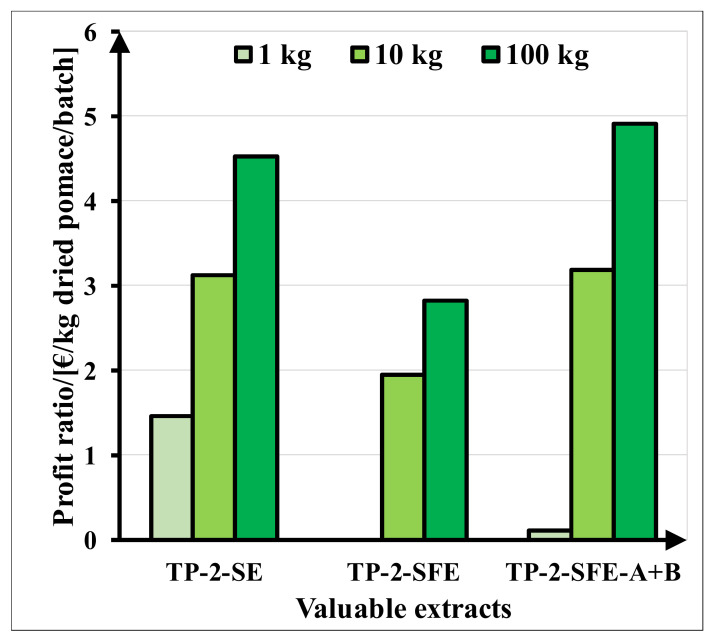
Profit ratio estimation for four valuable extracts (TP-2-SE, TP-2-SFE, TP-2-SFE-A and TP-2-SFE-B) and three scale-up capacities (1:10:100 kg dried pomace/batch).

For a small-capacity plant (1 kg dried pomace/batch), no profit is obtained for the supercritical CO_2_ extraction process. Increasing the processing capacity proportionally, the highest profit of 147.51 k€/y is calculated for the high-capacity plant (100 kg dried pomace/batch) and the SFE process with two products: pigmented solid oleoresin concentrated in lycopene (TP-2-SFE-B) and pigmented oil extract rich in carotenoids (TP-2-SFE-A). In Figure 9, we present the profit ratio (€/kg dried tomato pomace/batch) for three scale-up capacities.

For plants with small capacity (1 kg dried pomace/batch), the SE process is more profitable (profit ratio between 1.46 and 4.52 €/kg dried tomato pomace/batch). Increasing the processing capacity of the plant (100 kg dried pomace/batch), the SFE process becomes more profitable, especially for obtaining a lycopene-rich extract and pigmented oil (profit ratio 4.92 €/kg dried tomato pomace/batch).

## 3. Materials and Methods

### 3.1. Chemicals and Standards

Lycopene, β-carotene, 2,2-diphenyl-1-picrylhydrazyl (DPPH), 6-hydroxy-2,5,7,8-tetramethylbroman-2-carboxylic acid (Trolox), Folin–Ciocalteu reagent (2 N), gallic acid, anhydrous sodium carbonate, Supelco 37 Component FAME Mix, sodium hydroxide, boron trifluoride–methanol (BF_3_-MeOH) (10–14%) complex solution, anhydrous magnesium sulphate, acetone, ethyl acetate, methanol, bioethanol, n-heptane and hexane used in this study were of analytical grade, from Sigma-Aldrich (Munich, Germany). CO_2_ with 99.9% purity was acquired from Linde Gaz (Bucharest, Romania).

### 3.2. Preparation of Tomato Samples

Tomatoes of Rila variety, farmed in Colibași, Giurgiu County and harvested in June 2021 were purchased from a local farmer. Ripe tomatoes were cleaned of soil traces and were prepared to obtain tomato slices (TS), tomato pomace (TP) and tomato seeds (S). For TS (mixture of pulp, peels and seeds) preparation, tomatoes were manually cut into slices with a thickness of around 5 mm, while for TP (mixture of pulp residue, peels and seeds) preparation, tomatoes were manually pressed, using a squeezer (Ertone, model MN503, Aylesbury, UK) to remove the juice. For tomato seed (S) samples, the seeds were separated manually from the tomato pomace using a sieve. For all experiments, 245 kg of tomatoes with around 82 g of fresh TS (6 slices)/one tomato and 13 g of fresh TP/one tomato were used to prepare tomato samples. Samples were dried in a food dehydrator (Hendi Profi Line, model 229026, De Klomp, The Netherlands) at 50 °C for 48 h. Dehydration of tomato samples was conducted at this temperature because, at higher values, the degradation of carotenoid compounds proceeds faster [25]. Before extraction, tomato samples were ground using a grinder (Tarrington House, model KM150S, Bangalore, India) to decrease the particle size and improve the mass transfer during the extraction process.

### 3.3. Soxhlet Extraction Method

The Soxhlet extraction (SE) method includes four steps: preparation of tomato sample cartridge, extraction until the sample is saturated, separation of the solvent from the extract by evaporation and collection and storage of extract [44]. Here, 300 g of dehydrated and ground tomato samples (120 g of TS/120 g of TP/60 g of S) were used for SE extraction experiments. TS and TP samples were extracted for 6 h using two green solvents: bioethanol (1) and ethyl acetate (2). Tomato seed oil was extracted with hexane from S samples for 6 h. For each experiment, 20 g of sample and 250 mL of solvent was necessary. After extraction, the solvent was removed and recovered from the extract using a rotary evaporator (Hahnvapor, model HS-2000NS, Gimpo-si, Korea), while collected extracts were stored in the freezer at −20 °C until analysis. The results were expressed as g extract/100 g dried tomato sample.

### 3.4. Supercritical CO_2_ Extraction Method

Supercritical CO_2_ extraction (SFE) experiments were carried out in a laboratory-scale High-Pressure Extraction Unit (HPE-CC 500, Eurotechnica GmbH, Bargteheide, Germany). Here, 2160 g of dehydrated and ground tomato samples (1080 g of TP/1080 g of TS) was used for SFE experiments. For each experiment, 180 g of sample was loaded into the extractor and the air from the system was purged with CO_2_. Next, the extraction temperature was set and the extractor was pressurized with CO_2_ using the high-pressure CO_2_ pump at a desired value. The pressure and CO_2_ flow rate were monitored and kept constant using the pressure control valve and by adjusting the pump stroke. The CO_2_ flow rate was measured by the mass flow meter. During the experiment, the extract was collected every 30 min. The SFE experiments were performed at two sets of operating parameters. Set (1) was characterized by pressure of 400 bar, temperature of 70 °C, CO_2_ flow rate of 9 kg/h and extraction time of 10 h, while set (2) had higher values for pressure and CO_2_ flow rate as 450 bar and 11 kg/h, respectively. Collected extracts were stored in the freezer at −20 °C until analysis. Between all the experiments, the plant was washed with bioethanol to remove and recover extract traces from plant pipes. The results were expressed as g extract/100 g dried tomato sample.

### 3.5. Raw Extract Centrifugation Method

Oleoresins were centrifuged at 6000 rpm for 30 min (Hettich centrifuge, model EBA 200S, Hettich, Germany) to isolate different fractions rich in carotenoids and oil. The obtained fractions were separated, weighed and analyzed by UV–VIS, FT–IR and DPPH methods to determine their composition of carotenoids and antioxidant activities.

### 3.6. UV–VIS Qualitative and Quantitative Analysis of Carotenoids

Quali-quantitative analysis of tomato extracts was performed with the UV–VIS spectrometry method using a Helios UV–Visible spectrophotometer (Helios beta, Thermo Spectronic, Waltham, MA, USA). Spectra were recorded using acetone/hexane (*v*/*v*, 1:1) in the absorbance range of 0.3–0.9 [45] and in the wavelength range of 325–575 nm. Quantification of carotenoids was determined with the IPM-II-WG6 method, described in a previous study [33], using sample absorbances at 461 (isosbestic point) and 504 nm wavelengths. The results were expressed as mg of carotenoid/100 g of extract.

### 3.7. FT–IR Qualitative Analysis of Tomato Samples

Qualitative analysis of tomato samples was performed with the Fourier transform infrared spectroscopy method using a Bruker spectrometer (FT-IR Bruker Vertex 70, Ettlingen, Germany). Samples were analyzed using an ATR accessory with a diamond crystal. Spectra were collected in the spectral range of 4000–400 cm^−1^, at a spectral resolution of 4 cm^−1^ and 32 scans. Centrifuged fractions, tomato seed oil and pure carotenoids were placed directly onto the surface of the diamond crystal, ensuring full coverage, without any previous preparation of the samples. The ATR crystal was cleaned between samples. Differentiation of the samples was performed using the relative intensity of absorption bands and checking the presence of typical bands [46]. Fourteen major peaks characteristic of pure compounds, oil, water and ethanol’s typical bands were identified on recorded FT–IR spectra.

### 3.8. GC–MS Analysis of FAME Compounds

Tomato seed oil was subjected to a transesterification reaction to obtain FAMEs with an acid-catalyzed procedure, using BF_3_-MeOH complex as a catalyst. FAME preparation was performed in two stages, starting with sodium hydroxide saponification and followed by boron trifluoride-catalyzed esterification, according to the standard method [47]. The determination of FAME components in transesterified tomato seed oil was performed with the gas chromatography coupled to mass spectrometry (GC–MS) method, using a gas chromatograph (Agilent Technologies 7890A, Santa Clara, CA, USA) equipped with a mass spectrometer detector (Agilent Technologies 5975C), following the procedure described in a previous study [48]. FAME components were identified by comparing the retention time of each peak with those of a standard mixture of 37 fatty acid methyl esters subjected to GC–MS analysis under the same conditions. The results were expressed as the percentage of FAMEs in the oil (g/100 g oil).

### 3.9. Spectrophotometric Analysis of Antioxidant Activity

Total antioxidant activity of tomato extract samples was assessed using the DPPH (2,2-diphenyl-1-picrylhydrazyl) free radical scavenging assay [43] with some modifications. Briefly, 0.1 g of extract was vortexed at 2500 rpm for 5 min with 20 mL of methanol. Next, 400 µL of methanolic extract solution was mixed with 6 mL DPPH solution (0.1 mM) and incubated in the dark and at room temperature for 30 min. The absorbance at 517 nm was spectrophotometrically measured. Trolox (6-hydroxy-2,5,7,8-tetramethylbroman-2-carboxylic acid) was used as a reference antioxidant compound and the calibration curve was obtained in the 0–1 mM concentration range. The results were expressed as the percentage inhibition of free radicals, inhibition % DPPH.

### 3.10. Spectrophotometric Analysis of Total Phenolic Content 

Total phenolic content of tomato extract samples was evaluated with the Folin–Ciocalteu phenol reagent method [49] with some modifications. Briefly, 0.1 g of extract was vortexed at 2500 rpm for 5 min with 10 mL of methanol. Next, 0.5 mL of methanolic extract solution was mixed with 2.5 mL of Folin–Ciocalteu solution (10%) for 5 min, and 2 mL of sodium carbonate solution (7.5%) was added. The reaction mixture was incubated in the dark and at room temperature for 60 min, and the absorbance at 765 nm was spectrophotometrically measured. Gallic acid was used as a reference phenolic compound, and the calibration curve was obtained in the 10–80 µg/mL concentration range. The results were expressed as mg of gallic acid equivalents (mg GAE)/g of extract.

### 3.11. Economic Analysis

Economic analysis was performed considering four valuable products (TP-2-SE, TP-2-SFE, TP-2-SFE-A and TP-2-SFE-B) and three extraction capacities with ratio 1:10:100 kg of dried tomato pomace per batch, and the scale-up criterion was a constant extract mass/feed mass ratio [50]. The working time per batch was 24 h (including raw material grinding, extraction and cleaning), with 300 batches per year. The manufacturing costs (MCO, €/y) were calculated as the sum of equipment costs (CAPEX, €/y) and raw materials and utilities costs (OPEX, €/y). Raw material costs (RMCO) were related especially to solvent (ethyl acetate or CO_2_) costs. Their values were estimated considering the prices of industrial food-grade ethyl acetate between 5.6 and 18.7 €/kg depending on purchased quantity [51] and CO_2_ price as 2.11 €/kg [52]. Utilities costs (UCO) comprised thermal agents and the electricity used in the extraction process for organic solvent evaporation, pumping, heating and chilling. These costs were evaluated as a percentage of the operating costs (30% for SE process, 40% for SFE process and 45% for SFE + centrifugation process). For the SE process, operating costs comprised also the solvent recovery costs as 11–21%. Tomato pomace was considered free of charge, being acquired from the industrial preparation of tomato juice. Investment costs (CAPEX) were not considered in this analysis if there already existed extraction facilities with appropriate capacities, used for the extraction of other compounds. To estimate the revenue gained from products sold in the food and health market, the following prices were considered: 50 €/kg SE raw extract, 30 €/kg SFE raw extract, 150 €/kg SFE pigmented solid oleoresin concentrated in lycopene and 35 €/kg SFE pigmented oil extract rich in carotenoids. These prices were chosen according to the quality of each product and market price (111–666 €/kg lycopene extract obtained from tomatoes [53,54] and 65–233 €/kg pigmented oil obtained from tomatoes [55] through solvent extraction process).

### 3.12. Statistical Analysis

Extraction (Soxhlet and supercritical CO_2_ extraction) experiments, centrifugation of supercritical CO_2_ raw extracts from tomato samples and transesterification of tomato seed oil were performed in triplicate. Chemical analysis of tomato extracts for the identification and quantification of carotenoids, lycopene and β-carotene (UV–VIS and FT–IR methods), determination of FAME composition (GC–MS method), antioxidant activity (DPPH method) and total phenolic content analysis (Folin–Ciocalteu method) were carried out in triplicate. The results are reported as mean values ± standard deviation (SD). All experimental datasets were subjected to ANOVA analysis to determine the variability between samples and within samples [56]. The proposed null hypothesis for ANOVA analysis was that there was no statistically significant difference among the datasets, and the F ratio was calculated. By association of the F-test with *p*-value smaller than 0.05, the null hypothesis was rejected at a significance level of α = 0.05.

## 4. Conclusions

Lycopene and β-carotene are the main compounds in tomato extracts. Extraction yields, carotenoid contents and the quality of the extracts are influenced by the seeds’ contents, solvent affinity and polarity in the SE method and operating parameters in the SFE method. It was observed that a higher seed content of tomato pomace leads to increased extraction yields towards tomato slices, regardless of the extraction solvent in SE or operating parameters in SFE. Carotenoids are concentrated mostly in tomato peels, while tomato seeds act as a modifier, improving the extraction through their oil content. TP samples contain higher amounts of both peels and seeds compared to TS samples. Through the centrifugation step, it was found that raw extracts contain between 60 and 80% oil with dissolved carotenoids and 5–15% solid oleoresin concentrated in lycopene. The lycopene/β-carotene ratios in oily fractions are lower than in the solid oleoresins. β-carotene is predominant in oily fractions, and lycopene in solid oleoresin fractions. The superior quality of SFE towards SE extracts was shown in terms of lycopene and β-carotene content, antioxidant activity and the total phenolic content. Evaluating the economic profitability of plants with three scale capacities, the feasibility increases for capacities greater than 100 kg of dried pomace/batch for the SFE process, through which two valuable products are obtained, lycopene-rich and pigmented oil extracts, due to the higher price of these products on the market. For the SE plant, profit can be gained even on a small-capacity plant (1 kg dried pomace/batch).

Extracts obtained from tomatoes with green solvents such as bioethanol, ethyl acetate and supercritical carbon dioxide have high carotenoid content and can be used in the food, pharmaceutic and cosmetic industries due to their quality. The supercritical fluid extraction method at 450 bar, 70 °C and 11 kg/h flow rate using green CO_2_ is recommended for both tomato samples due to the superior quality of the recovered extract and the positive impact on the environment and human health. Higher profitability is gained for a plant capacity over 100 kg.

## Figures and Tables

**Table 1:** Solvent types and polarity [27].

Solvent	Green Solvent	Solvent Polarity
Water	✓	1.000
Ethanol	✓	0.654
Acetone	✓	0.355
Dichloromethane	✗	0.309
Ethyl Acetate	✓	0.228
Chloroform	✗	0.052
Hexane	✗	0.009
Vegetable Oils	✓	0.000
Carbon Dioxide	✓	0.000

**Table 2:** Preparation of tomato samples: experimental data.

Sample Type	Tomato Mass (kg)	Tomato Number (Pieces)	Juice Mass (kg)	Fresh Sample Mass (kg)	Dried Sample Mass (kg)
Tomato slices (TS)	18.7	220	-	18.7 (1363 slices)	1.2
Tomato pomace (TP)	187.0	1460	165	19.0	1.2
Tomato seeds (S)	39.0	305	35	4.0	0.06

**Table 3:** Extraction yields of tomato samples (g extract/100 g dried tomato sample ± SD).

Extract ID	Extraction Method	Sample Type/ Extraction Solvent	Extraction Parameters *	Extract Type	Extract Consistency	Extraction Yield **
TSO-SE	SE	Tomato Seeds/ Hexane	m_sample_ = 20 g V_solvent_ = 250 mL	Raw extract	Oil	20.21 ± 1.22 ^a^
TS-1-SE	SE	TomatoSlices/ Bioethanol	m_sample_ = 20 g V_solvent_ = 250 mL	Raw extract	Solid oleoresin	5.92 ± 0.69 ^b^
TS-2-SE		Tomato Slices/ Ethyl Acetate	m_sample_ = 20 g V_solvent_ = 250 mL	Raw extract	Solid oleoresin	8.72 ± 0.93 ^b^
TP-1-SE		Tomato Pomace/ Bioethanol	m_sample_ = 20 g V_solvent_ = 250 mL	Raw extract	Solid oleoresin	13.23 ± 1.14 ^c^
TP-2-SE		Tomato Pomace/ Ethyl Acetate	m_sample_ = 20 g V_solvent_ = 250 mL	Raw extract	Solid oleoresin	14.33 ± 1.19 ^c^
TS-1-SFE	SFE	Tomato Slices/ Supercritical CO_2_	m_sample_ = 180 g T = 70 °C P = 400 bar G_CO2_ = 9 kg/h	Raw extract	Liquid oleoresin	5.25 ± 0.79 ^b^
TS-1-SFE-A				Fraction A	Oil	3.83 ± 1.28 ^b^
TS-1-SFE-B				Fraction B	Solid oleoresin	0.35 ± 0.02 ^b^
TS-1-SFE-C				Fraction C	Liquid	1.07 ± 0.26 ^b^
TS-2-SFE			m_sample_ = 180 g T = 70 °C P = 450 bar G_CO2_ = 11 kg/h	Raw extract	Liquid oleoresin	6.64 ± 1.12 ^b^
TS-2-SFE-A				Fraction A	Oil	4.38 ± 0.86 ^b^
TS-2-SFE-B				Fraction B	Solid oleoresin	0.30 ± 0.03 ^b^
TS-2-SFE-C				Fraction C	Liquid	1.97 ± 0.09 ^b^
TP-1-SFE	SFE	Tomato Pomace/ Supercritical CO_2_	m_sample_ = 180 g T = 70 °C P = 400 bar G_CO2_ = 9 kg/h	Raw extract	Liquid oleoresin	10.02 ± 1.14 ^c^
TP-1-SFE-A				Fraction A	Oil	7.69 ± 1.01 ^c^
TP-1-SFE-B				Fraction B	Solid oleoresin	1.27 ± 0.19 ^c^
TP-1-SFE-C				Fraction C	Liquid	1.06 ± 0.04 ^c^
TP-2-SFE			m_sample_ = 180 g T = 70 °C P = 450 bar G_CO2_ = 11 kg/h	Raw extract	Liquid oleoresin	12.35 ± 1.55 ^c^
TP-2-SFE-A				Fraction A	Oil	8.95 ± 1.07 ^c^
TP-2-SFE-B				Fraction B	Solid oleoresin	1.85 ± 0.11 ^c^
TP-2-SFE-C				Fraction C	Liquid	1.57 ± 0.12 ^c^

* m_sample_/[g]—the mass of the sample, V_solvent_/[mL]—the volume of the solvent, T/[°C]—the extraction temperature, P/[bar]—the extraction pressure, G_CO2_/[kg/h]—the solvent flow rate. ** means ± SD followed by a letter (a–c) indicate that there are no statistically significant differences between extraction yields with the same superscript letter according to Hartley’s F_max_ test (*p* < 0.05) at level of significance α = 0.05.

**Table 4:** FT–IR spectral bands identified for pure carotenoids and tomato samples.

Peak ID	Pure Lycopene	Pure β-Carotene	TP-2-SFE-A	TP-2-SFE-B	TP-2-SFE-C	TSO-SE
1	-	-	-	-	3240–3360	-
2	3032–3037	-	3006–3010	3000–3016	-	3006–3010
3	-	-	-	-	2977–2986	-
4	2910–2915	2895–2923	2919–2925	2919–2925	-	2920–2925
5	2846–2859	2845–2862	2852–2855	2850–2854	-	2852–2855
6	-	-	1742–1745	1741–1745	-	1741–1745
7	1627–1631	-	-	-	1628–1652	-
8	1438–1445	1441–1444	1458–1465	1459–1465	-	1459–1464
9	1361–1375	1364–1370	1370–1381	1370–1381	-	1371–1380
10	-	-	1227–1244	1235–1238	-	1225–1245
11	-	-	1157–1164	1157–1164	-	1154–1165
12	1097–1107	-	1093–1102	1091–1104	-	1094–1102
13	-	-	-	-	1043–1047	-
14	957	964–965	963–973	958–974	-	963–971

**Table 5:** FAME composition of tomato seed oil.

Extract ID	FAME Profile/[%] *			
	Palmitic Acid (C16:0)	Stearic Acid (C18:0)	Oleic Acid (C18:1)	Linoleic Acid (C18:2)
TSO-SE	15.08 ± 0.01 ^a^	6.03 ± 0.43 ^b^	23.41 ± 0.56 ^b^	55.59 ± 0.12 ^a^

* means ± SD followed by a letter (a, b) indicate that there are no statistically significant differences between fatty acid compositions with the same superscript letter according to Hartley’s Fmax test (*p* < 0.05) at α = 0.05 level of significance.

**Table 6:** Total phenolic content (mg GAE/g extract) ± SD and DPPH antioxidant activity (% inhibition ± SD) of tomato samples.

Extract ID	Total Phenolic Content *	Antioxidant Activity *
TS-1-SE	26.93 ± 0.12 ^a^	43.35 ± 3.36 ^b^
TS-2-SE	17.32 ± 0.08 ^a^	27.61 ± 1.26
TP-1-SE	30.91 ± 0.14 ^a^	40.78 ± 3.41 ^c^
TP-2-SE	25.25 ± 0.11 ^a^	34.46 ± 2.04 ^c^
TS-1-SFE	22.59 ± 0.06 ^a^	47.59 ± 4.01 ^b^
TS-1-SFE-A	-	41.03 ± 3.15 ^b^
TS-1-SFE-B	-	29.89 ± 2.05 ^b^
TS-2-SFE	33.17 ± 0.12 ^a^	40.05 ± 3.04 ^b^
TS-2-SFE-A	-	49.65 ± 4.21 ^b^
TS-2-SFE-B	-	58.85 ± 4.09 ^b^
TP-1-SFE	27.73 ± 0.11 ^a^	39.26 ± 3.02 ^c^
TP-1-SFE-A	-	42.39 ± 4.04 ^c^
TP-1-SFE-B	-	54.46 ± 4.17 ^c^
TP-2-SFE	35.25 ± 0.14 ^a^	37.30 ± 3.25 ^c^
TP-2-SFE-A	-	38.41 ± 3.04 ^c^
TP-2-SFE-B	-	67.02 ± 5.11 ^c^

* means ± SD followed by a letter (a–c) indicate that there are no statistically significant differences between total phenolic content and antioxidant activity with the same superscript letter according to Hartley’s F_max_ test (*p* < 0.05) at α = 0.05 level of significance.

**Table 7:** Estimated values for annual manufacturing cost components.

Capacity (kg Dried Pomace/Batch)	Extraction Products	Production (kg Extract/y)	Revenue (k€/y)	MCO = OPEX (k€/y)	RMCO (% from OPEX)	UCO (% from OPEX)	Solvent Recovery (% from OPEX)	Profit (k€/y)
1	TP-2-SE	42.99	2.15	1.71	59.32	30.00	10.68	0.44
	TP-2-SFE	37.05	1.11	1.59	60.00	40.00	-	0.00
	TP-2-SFE-A	26.85	0.94	1.73	55.00	45.00	-	0.03
	TP-2-SFE-B	5.49	0.82					
10	TP-2-SE	429.90	21.50	12.13	55.78	30.00	14.22	9.37
	TP-2-SFE	370.50	11.12	5.28	60.00	40.00	-	5.83
	TP-2-SFE-A	268.50	9.40	8.07	55.00	45.00	-	9.56
	TP-2-SFE-B	54.90	8.24					
100	TP-2-SE	4299.00	214.95	79.25	49.51	30.00	20.49	135.70
	TP-2-SFE	3705.00	111.15	26.42	60.00	40.00	-	84.73
	TP-2-SFE-A	2685.00	93.98	28.82	55.00	45.00	-	147.51
	TP-2-SFE-B	549.00	82.35					

## Data Availability

Data are available by the corresponding author upon request.

## Sample Availability

Samples of the compounds are not available from the authors.
